# Prevalence of syphilis diagnosed in female inmates of city Ponta Porã

**DOI:** 10.1186/1753-6561-8-S4-P81

**Published:** 2014-10-01

**Authors:** Rafael Henrique Oliveira Lopes, Isabella Jacó Carrijo, Maisa Estopa Correa, Marco Antonio Moreira Puga, Ana Rita Coimbra Motta Castro, Sandra Maria do Valle Leone Oliveira, Julio Henrique Rosa Croda, Grazielli Rocha Rezende, Larissa Melo Bandeira, Everton Ferreira Lemos, Simone Simionatto

**Affiliations:** 1 Federal University of Grande Dourados, Dourados, Mato Grosso do Sul,Brazil; 2 Infectious Diseases, Federal University of Mato Grosso do Sul, Dourados, Brazil; 3 Federal University of Mato Grosso do Sul, Dourados, Brazil; 4 Faculty of Health Sciences, Federal University of Grande Dourados, Dourados, Brazil

## Background

Syphilis is a growing public health problem in several countries. The infection is systemic, usually involving mucocutaneous ulcers and rashes in the early phases, and a range of serious complications including cardiovascular and neurological disease in later phases [1,2]. Global control of syphilis is hampered by slow and insensitive diagnostic methods, particularly for risk population like prison inmates [3,4]. This study aimed to analyze the prevalence and socio-demographic, behavioral and institutional factors associated with *Treponema pallidum infection *in prison women in Ponta Porã city, Mato Grosso do Sul.

## Methods

The study was conducted from January to September 2013 and the sample size was calculated based on the prevalence of syphilis in Brazil. The sample was randomly selected and included 74 female inmates in Ponta Porã. To determine the prevalence of T*reponema pallidum infection*, blood samples were collected for serological tests using a treponemal test named enzyme-linked immunosorbent assay (ELISA) and a non-treponemal test e venereal disease research laboratory (VDRL). Each test was performed in accordance with manufacturer's recommendations. Socio-demographic and clinical information, as well as variables related to transmission were collected in a standard questionnaire. Research Electronic Data Capture (REDCap) is being carried out to store data. The study was approved by the Research Ethics Committee of the Universidade Federal da Grande Dourados, Brazil.

## Results

During the study, 11 cases of *Treponema pallidum *infection were identified, which represents a prevalence of 14.8 %. The serology analyses showed that the prevalence was higher than other studies, where the prevalence ranged from 0.5% and 7.5% [5]. The risk factors are still being evaluated by REDCap. Different studies have shown an increased vulnerability of the incarcerated population to syphilis, associated to other factors like high-risk sexual behavior. The quality of incarcerated women's life could be improved by routine infection diagnosis, the implantation of a screening program for the health problems and systematic education.
